# Enhanced Membrane Pore Formation through High-Affinity Targeted Antimicrobial Peptides

**DOI:** 10.1371/journal.pone.0039768

**Published:** 2012-06-29

**Authors:** Christopher J. Arnusch, Roland J. Pieters, Eefjan Breukink

**Affiliations:** 1 Department of Membrane Biochemistry and Biophysics, Utrecht University, Utrecht, The Netherlands; 2 Department of Medicinal Chemistry and Chemical Biology, Utrecht University, Utrecht, The Netherlands; University of Melbourne, Australia

## Abstract

Many cationic antimicrobial peptides (AMPs) target the unique lipid composition of the prokaryotic cell membrane. However, the micromolar activities common for these peptides are considered weak in comparison to nisin, which follows a targeted, pore-forming mode of action. Here we show that AMPs can be modified with a high-affinity targeting module, which enables membrane permeabilization at low concentration. Magainin 2 and a truncated peptide analog were conjugated to vancomycin using click chemistry, and could be directed towards specific membrane embedded receptors both in model membrane systems and whole cells. Compared with untargeted vesicles, a gain in permeabilization efficacy of two orders of magnitude was reached with large unilamellar vesicles that included lipid II, the target of vancomycin. The truncated vancomycin-peptide conjugate showed an increased activity against vancomycin resistant *Enterococci,* whereas the full-length conjugate was more active against a targeted eukaryotic cell model: lipid II containing erythrocytes. This study highlights that AMPs can be made more selective and more potent against biological membranes that contain structures that can be targeted.

## Introduction

Antimicrobial peptides (AMPs) are crucial components of the innate immune system that act predominantly at the level of the plasma membrane of the bacterial cell [1], [2]. Many AMPs have both antimicrobial and anticancer activity, since membranes of cancerous eukaryotic cells share features with those of prokaryotic cells, i.e. a relatively high negative charge [3]–[5]. The magainin peptides were discovered early and became the paradigm for the mode of action for many cationic amphipathic AMPs [6], [7]. However, this group of polypeptides lacks sufficient affinity for its target membranes, and activity is commonly observed at micromolar concentrations. This limits their medicinal use because the effective dose is close to the toxic dose [8]. Increasing the activity, or reducing the toxicity of AMPs may broaden this therapeutic window and this is essential for the application of these peptides in combating the growing resistance of antimicrobial agents to pathogens, and may complement or enhance existing anticancer therapies.

The mechanisms of action of AMPs in general have been studied in great detail [9]–[11]. AMPs that are active against eukaryotic cells may potentially be used in anticancer therapy, although they also suffer from low selectivity and high toxicity. For the magainins and other cationic AMPs, anionic structures in cancer cell plasma membranes such as the lipid phosphatidyl serine [12], and O-glycosylated mucins [13] may be responsible for their attraction to the eukaryotic cell, and their cell permeabilization activity [14]. However, a dramatic improvement of the high micromolar activities requires a targeted mode of action for cationic AMPs.

The nisin-lipid II system is to the best of our knowledge the most active pore forming peptide system known. The first step of nisin’s targeted pore forming mechanism involves the N-terminal part of nisin, which specifically binds the pyrophosphate part of the lipid II molecule [15]–[19]. Subsequently, C-terminus membrane insertion and pore assembly occurs, which causes leakage of the cell content. This process is 1000 times more efficient than AMPs that rely predominantly on an electrostatic attraction to the cell membrane [20]. Here we aimed to mimic the highly effective targeted pore forming mechanism observed in nisin by conjugation of a high-affinity targeting moiety to an AMP. Such a system could potentially be adapted to target various types of cells, depending on the targeting moiety.

**Figure 1:**
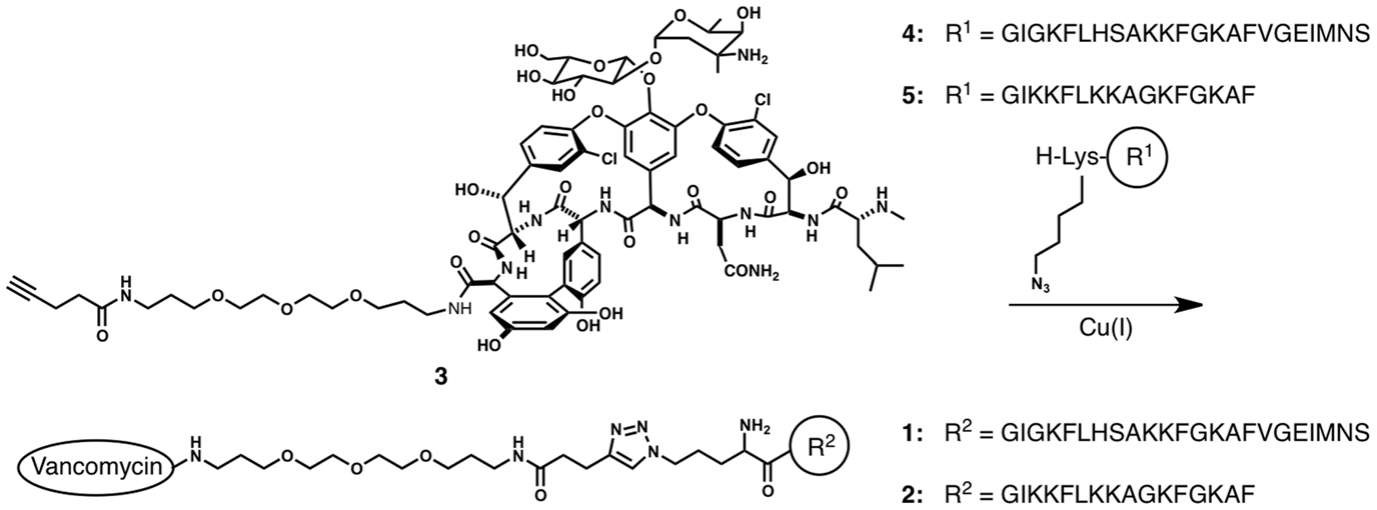
Synthesis of the vancomycin-targeted AMPs.

We focused to increase the membrane disrupting potency of two peptides based on the magainin 2 sequence by linking a targeting moiety (Figure 1). Vancomycin was chosen for targeting, since it binds lipid II. As for the AMPs, we chose the full-length magainin 2 sequence, and compared it to a shorter optimized analog sequence [21]. We observed a large targeting effect on large unilamellar vesicles (LUVs) that contained lipid II. The conjugates showed decreased activity with vancomycin susceptible bacterial strains compared to vancomycin alone, but showed an increased antimicrobial activity against vancomycin resistant *Enterococci* with the shorter vancomycin-peptide conjugate. Strikingly, on eukaryotic cells, the full-length vancomycin-peptide conjugate caused permeabilzation of erythrocytes that contained lipid II with a remarkably potency similarly to nisin. This result broadens the applicability of targeted AMPs towards anticancer therapy and shows that permeabilization of any eukaryotic cell of choice may be possible, provided that the AMP can be specifically targeted to the cell membrane.

## Results and Discussion

We synthesized the conjugates using the highly efficient CuAAC click chemistry. This reaction is the copper-mediated coupling between an azide and an alkyne, and has been used in numerous applications [22]. We coupled lysine bearing an azide on its side chain to the N-terminus of these peptides on the solid phase [23]. Vancomycin was functionalized with an alkyne functional group as previously reported [24]. Conjugation was achieved upon dissolving the functionalized precursors in an aqueous solution with addition of sodium ascorbate, CuSO_4_ and heating in the microwave. Both conjugates were purified using HPLC (>98%, Figure S1) and identified using mass spectrometry.

**Figure 2:**
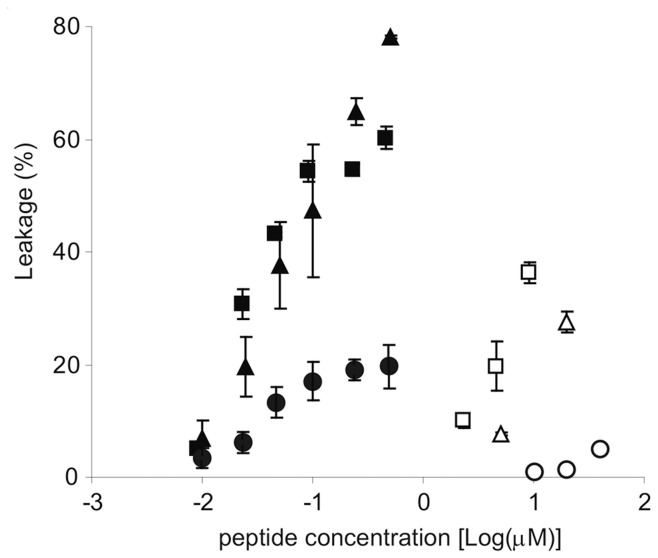
Large unilamellar vesicles (25 µM) consisting of (DOPC/DOPE/SM) (46/12/42) 60%, cholesterol (40%), and lipid II (0.1%), which contained a solution of carboxyfluorescein were treated with 1 (▪), 2 (•) and nisin (▴). Vesicles without lipid II were treated with **1** (□), **2** (○) and magainin 2 (▵).

In order to test if vancomycin was indeed a good targeting module for the peptide, the leakage of carboxyfluorescein was measured from LUVs with a lipid composition that represented the outer leaflet of the eukaryotic cell (60% DOPC:SM:DOPE (46∶42:12), 40% cholesterol), and compared this to similar vesicles containing 0.1% lipid II. This lipid composition would give a clear indication of the targeting ability of the vancomycin portion since the cationic peptide would not be strongly attracted to the zwitterionic head groups (phosphatidylcholine, and sphingomyelin). In this system, nisin caused leakage in vesicles that contained lipid II at nanomolar concentrations, whereas vesicles that did not contain lipid II required much higher concentrations (Figure 2). We found that the vancomycin-magainin construct **1** caused leakage with a similar potency to nisin, i.e. at low nanomolar concentrations. Similarly, the shorter conjugate **2** caused lipid II dependent leakage, although only a maximum of 20% leakage could be achieved. Control experiments with unfunctionalized magainin peptides tested on vesicles with or without lipid II, or the targeted peptides and nisin tested on vesicles that did not contain lipid II, all caused carboxyfluorescein leakage at the expected micromolar concentrations. Previously reported data showed that magainin 2 does not depend on lipid II content [19]. Our results suggest that the length of the peptide may be important in this vesicle system, which mimics the eukaryotic cell membrane. Previous studies on similar AMPs of different length show that this parameter is significant for the mode of action [25]. Shorter peptides may tend to follow the carpet model [26] whereas longer peptides at least have the potential for transmembrane orientation. Compound **1**, consisting of a peptide (24 amino acids), vancomycin (7 amino acids) and a linker is approximately the same length as nisin (34 amino acids). The shorter conjugate **2** gives significantly less leakage. By conjugating magainin to a specific targeting structure in this system, an improvement of approximately 100 fold was obtained.

Next the conjugates were tested on a small series of Gram-positive bacteria including methicillin resistant *S. aureus*, (MRSA) and vancomycin susceptible, and resistant *Enterococci* (VSE, and VRE respectively) (Table 1). For the vancomycin-magainin conjugates **1** and **2**, no advantage was seen with the vancomycin susceptible Gram-positive strains. Vancomycin-lipid II binding was probably slightly perturbed due to peptide modification, thus compared to native vancomycin, a weaker inhibition was seen. Other possibilities for reduced potency include the peptide interaction with the negatively charged structures in the cell wall such as the teichoic acids, or peptide aggregation within the cell wall decreased accessibility of the vancomycin part to its target, and negatively affected its potency. Interestingly, a significant gain in activity was observed with conjugate **2** with VRE. In this case, the contribution of the vancomycin component is much weaker, revealing the effect of the peptide portion of the conjugate. In contrast to the susceptible strains, peptide interaction with the cell wall or membrane may lead here to increased concentrations leading to an activity advantage. This effect may be comparable to other glycopeptide analogs that contain a hydrophobic anchor to target the membrane (oritavancin, televancin, dalbavancin). Resistance to vancomycin (vanA or vanB type resistance) due to a substitution of D-Lac for the terminal D-Ala residue results in a decreased level of vancomycin binding and therefore decreased efficacy. The N-terminal portion of nisin, nisin(1-12), should not be susceptible to this mutation since it binds the pyrophosphate portion of lipid II. We therefore synthesized a nisin(1-12)-compound **5** conjugate and observed that this conjugate gave a weaker activity (32 µg/mL) than compound **2** (16 µg/mL) (see Methods S1, and Table S1). This is probably due to the relatively weak binding ability of nisin(1-12) alone to Lipid II: Nisin(1-12) alone gave activities of 128 µg/mL for both VSE and VRE.

**Table 1:** Minimum inhibitory concentration (MIC, µg/mL) of vancomycin-magainin peptide derivatives.

Compound^a^	V^b^	4	1	5	2
MRSA^c^ (15A761)	0.75	>256	32	256	2
MRSA (15A763)	0.4	>256	32	256	2
VSE^d^ (15A797)	0.5	256	16	128	4
VRE^e^ (15A799)	128	256	256	64	16
*M. catarhallis* (58L028)	32	64	64	8	32

a compounds tested in conjunction with compounds reported in ref. [24] ^b^V: vancomycin, values first published in ref. [24], ^c^MRSA: methicillin resistant *S. aureus, ^d^*VSE: vancomycin susceptible *Enterococci, ^e^VRE:* vancomycin resistant *Enterococci.*

This growth inhibition assay gave a good indication of the biological activity in a living prokaryotic model system, although more VRE isolates should be tested to validate the activity increase of compound **2**. In addition, a more in-depth study would be needed to fully understand the mechanism of action in this biological system and rule out any additive effects the two components may have in combination. This may include experiments such as the checkerboard assay to determine the sum of the fractional inhibitory concentrations (ΣFIC), and would indicate the degree of synergy between the targeting component and the peptide component. This could be compared to a virtual ΣFIC calculated using the minimum inhibitory concentration (MIC) of the hybridized product for the contribution of the covalent linkage. A virtual ΣFIC calculated using compound **2,** and its two components was 0.19, giving a strong synergistic effect (see Methods S1). Note that in our assay, we report values with units µg/mL. Considering that the molecular weight of the hybridized product is approximately twice that of its two components, the observed gain of activity on a per molecule basis is more pronounced. As a control experiment, the compounds were also tested on a Gram-negative strain, *M. catarhallis*. As expected, no advantage was seen. Vancomycin is active only on Gram-positive bacteria and needs lipid II or nascent peptidoglycan as accessible targets to prevent crosslinking in the growing cell wall. Overall, these results show that access to the plasma membrane may be inhibited. However, access to the plasma membrane is not relevant with eukaryotic cells, which do not have cell walls.

**Figure 3:**
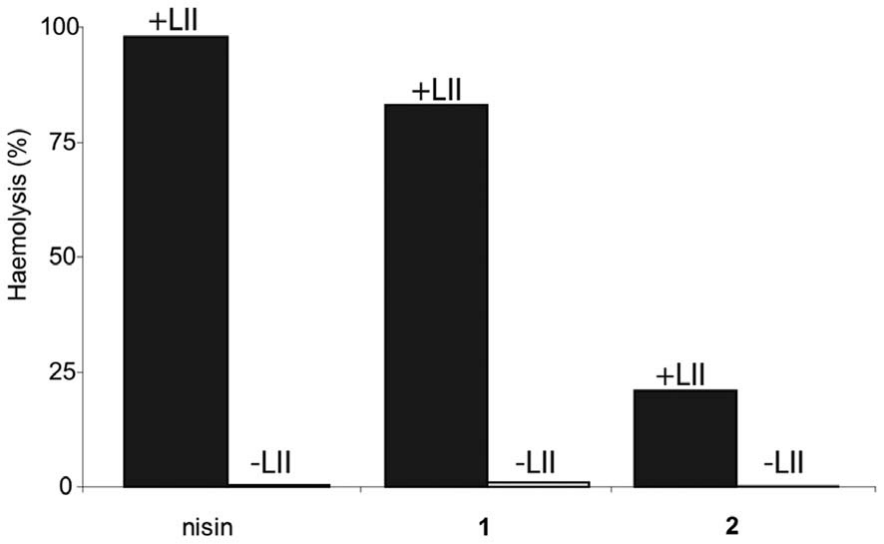
Amount of hemolysis from erythrocytes with and without lipid II, after treatment with nisin (10 µM) compound 1 (10 µM), and compound 2 (50 µM).

Finally, to test if the vancomycin-peptide conjugates were active towards a eukaryotic model system, we doped erythrocytes with a small amount of lipid II. This cell wall precursor is amphiphilic, and prefers to be incorporated in any membrane. Lipid II was incorporated into the membrane of human erythrocytes by rapidly mixing a solution of the erythrocytes in PBS with an ethanolic solution of lipid II. The peptide conjugates were then added and hemolysis was observed by measuring the release of hemoglobin into the solution. In this experiment, hemolysis would indicate a strong targeting effect. Indeed, strongly enhanced hemolysis was seen by the vancomycin-magainin conjugate **1** and by nisin with the lipid II containing erythrocytes (Figure 3 and Figure S2). For nisin, the maximum amount of hemolysis was 90–100%, which occurred between 5–10 µM. Similarly, for the vancomycin-magainin conjugate **1** the maximum hemolysis was between 60-80%, and occurred between 5-10 µM. In comparison, we observed no hemolysis with erythrocytes that did not contain lipid II, and no hemolysis of untargeted magainin at concentrations up to 100 µM. The shorter conjugate **2** showed a lipid II-dependent activity, however only a maximum hemolysis of ca. 20% could be reached.

### Conclusion

The addition of a targeting moiety can lead to enhance pore formation of model membranes, but this depends on the nature of the AMP [27]. The remarkable properties of the targeted peptide systems described here, namely, enhanced leakage with model membranes, bacterial growth inhibition, and notable activity towards a eukaryotic membrane reveals the versatility of such peptide conjugates. The latter ability was observed with potency comparable to nisin. This result shows the broader potential of cationic AMPs such as magainin linked to high-affinity targeting modules as potential therapeutics in anticancer applications where both increased cell selectivity and high potency are crucial.

## Materials and Methods

### Antibacterials and Reagents

Vancomycin was obtained from Sigma (The Netherlands). Nisin was purified from Chrisin®, which was generously donated from Christian Hansen (Denmark). Electrospray ionization (ESI) mass spectrometry was carried out using a Shimadzu LCMS QP-8000 single quadrupole bench top mass spectrometer (m/z range <2000), coupled with a QP-8000 data system. Analytical HPLC was performed on a Shimadzu Class-VP automated HPLC using an analytical reversed-phase column (Alltech Adsorbosphere C18, 300 Å, 5 µ m, 250×4.6 mm) and a UV detector operating at 220 nm and 254 nm. Preparative HPLC was performed on a Gilson automated HPLC using a preparative reversed-phase column (Alltech Adsorbosphere C18, 10 µ m, 250×22 mm) and a UV detector operating at 220 nm and 254 nm. Elution was effected using an appropriate gradient from 0.1% TFA in CH_3_CN/H_2_O (5/95, v/v)(buffer A) to 0.1% TFA in CH_3_CN/H_2_O (95/5, v/v) (buffer B) using a flow rate of 1 mL min.^−1^ (analytical) or 11.5 mL min.^−1^ (preparative). Microwave reaction was performed in an Initiator reactor from Biotage. Fluorescence measurements were performed on an SLM-Aminco SPF-500 C fluorimeter.

#### Vancomycin-magainin conjugate (1)

To compound **3** (2.2 mg, 1.25 µmol) was added H_2_O/CH_3_CN 1/1 (600 µL). This was added to **4** (4 mg, 1.25 µmol) and subsequently, CuSO_4_.5H_2_O (2.5 mg, 0.01 mmol) and NaAsc (2 mg, 0.01 mmol). The solution was mixed and gently heated until all components had dissolved, and the mixture was subject to microwave heating for 5 min. at a constant temperature of 80°C. The solvents were removed *in vacuo* and the crude mixture was subject to preparative HPLC. **1** was purified and lyophilized for 3.3 mg of a white powder in a yield of 53%. The identity of **1** was confirmed with MS. HPLC Retention time  = 17.1 min. >98%; MS *m/z*: 1088.4 [M +4H]^4+^, 1451.2 [M +3H]^3^.

#### Vancomycin-peptide conjugate (2)

Compound **3** (6 mg, 3.1 µmol) and peptide-azide **5** (6.3 mg, 2.4 µmol) were dissolved in H_2_O/DMF 18/1 (950 mL). CuSO_4_.5H_2_O (3 mg, 0.012 mmol) was added and mixed until dissolved, and NaAsc (6.1 mg, 0.03 mmol) was added and mixed until dissolved. The mixture was subject to microwave heating for 5 min. at a constant temperature of 80°C. Buffer A was added (1 mL) and the solution was subject to preparative HPLC. **2** was purified and lyophilized for 6.5 mg of a white powder in a yield of 59%. The identity of **2** was confirmed with MS. HPLC Retention time  = 15.7 min. >98%; MS *m/z*: 1826.8 [M +2H]^2+^.

#### Bacterial strains

Methicillin-resistant *Staphylococcus aureus* MRSA (15A761, and 15A763), vancomycin susceptible *Enterococci* (VSE, strain 15A797), and vancomycin resistant *Enterococci* (VRE, strain 15A799) were clinical blood isolates obtained from the strain collection of the University Medical Center (Utrecht, The Netherlands) from a teaching hospital in Coimbra, Portugal. *M. catarrhalis* (58L028) was obtained from Porto, Portugal.

### Antibacterial Activity

MICs were determined by broth microdilution, utilizing CA-MHB as described by the CLSI guidelines [National Committee for Clinical Laboratory Standards 2002. 5th ed., M7-A5.] (formerly the NCCLS guidelines). Experiments were performed in triplicate and the mean is reported.

#### 5(6)-Carboxyfluorescein leakage

Experiments were performed as previously described [19], [28]. The lipid composition used was 60% DOPC:SM:DOPE (46∶42:12), 40% cholesterol and 0.1% lipid II. In short, large unilamellar vesicles (LUV’s) containing 5-6-carboxyfluorescein were made by extrusion of multilamellar vesicles 10x through a polycarbonate membrane filter (0.2 µm) in 5-6-carboxyfluorescein (50 mM, 10 mM Tris pH 7, 150 mM NaCl). The LUV’s were separated from the extravesicular 5-6-carboxyfluorescein solution using Sephadex G50. The lipid concentration in the LUV’s was determined after destruction of the vesicles and Pi measurement. The change in fluorescence (excitation 492 nm, emission 515 nm) was measured upon addition of differing concentrations of antibiotic to the vesicles for a period of 3 minutes. The percent leakage was determined at 80 seconds, and the vesicles were destroyed with Triton-X100 detergent for maximum obtainable leakage at 160 seconds. Mean values including standard deviation of 3 measurements are reported.

#### Lipid II dependant hemolysis

Lipid II (7.5 µL, 0.04 mM in ethanol) was added to human red blood cells (hRBC, 1 mL, 1% suspension in phosphate buffered saline (PBS)) while gently vortexing. To 100 µL of this suspension was added the antibiotic, and incubated for 20 min. at 37°C with gentle shaking. The suspension was diluted with PBS (300 µL), centrifuged, and the absorbance of the supernatant was measured at 405, and 540 nm. Controls consisted of the preparation of the hRBC similar to above but addition of 7.5 µL ethanol/1 mL, 1% suspension in PBS buffer. Values for 0% and 100% hemolysis were obtained using hRBC (100 µL) suspended in PBS, incubated as above, diluted with PBS (300 µL) (0% hydrolysis), or with water (300 µL) (100% hemolysis). Experiments were performed in duplicate and the mean is reported.

## Supporting Information

Figure S1 **HPLC traces of vancomycin-peptide conjugates.**
(TIF)

Figure S2 Targeted hemolysis. Erythrocytes containing lipid II (0.1%), were treated with 1 (▵), 2 (○), nisin (□), 4 (*) and 5 (⋄). Vesicles without lipid II were treated with **1** (▴), 2 (•), nisin (▪), 4 (**×**), 5 (⧫), and vancomycin (**+**).
(TIF)

Methods S1 **ΣFIC calculations for compound 2 and VRE, and synthesis of the Nisin(1-12)-peptide conjugate.**
(DOC)

Table S1 **Minimum inhibitory concentration (MIC, µg/mL) of Nisin(1-12)-peptide derivatives.**
(DOCX)
