# Supplementary figures and images for: Enhanced Membrane Pore Formation through High-Affinity Targeted Antimicrobial Peptides
Source: PLoS One. 2012 Jun 29;7(6):e39768. doi: 10.1371/journal.pone.0039768 (PMC3387250; doi:10.1371/journal.pone.0039768)

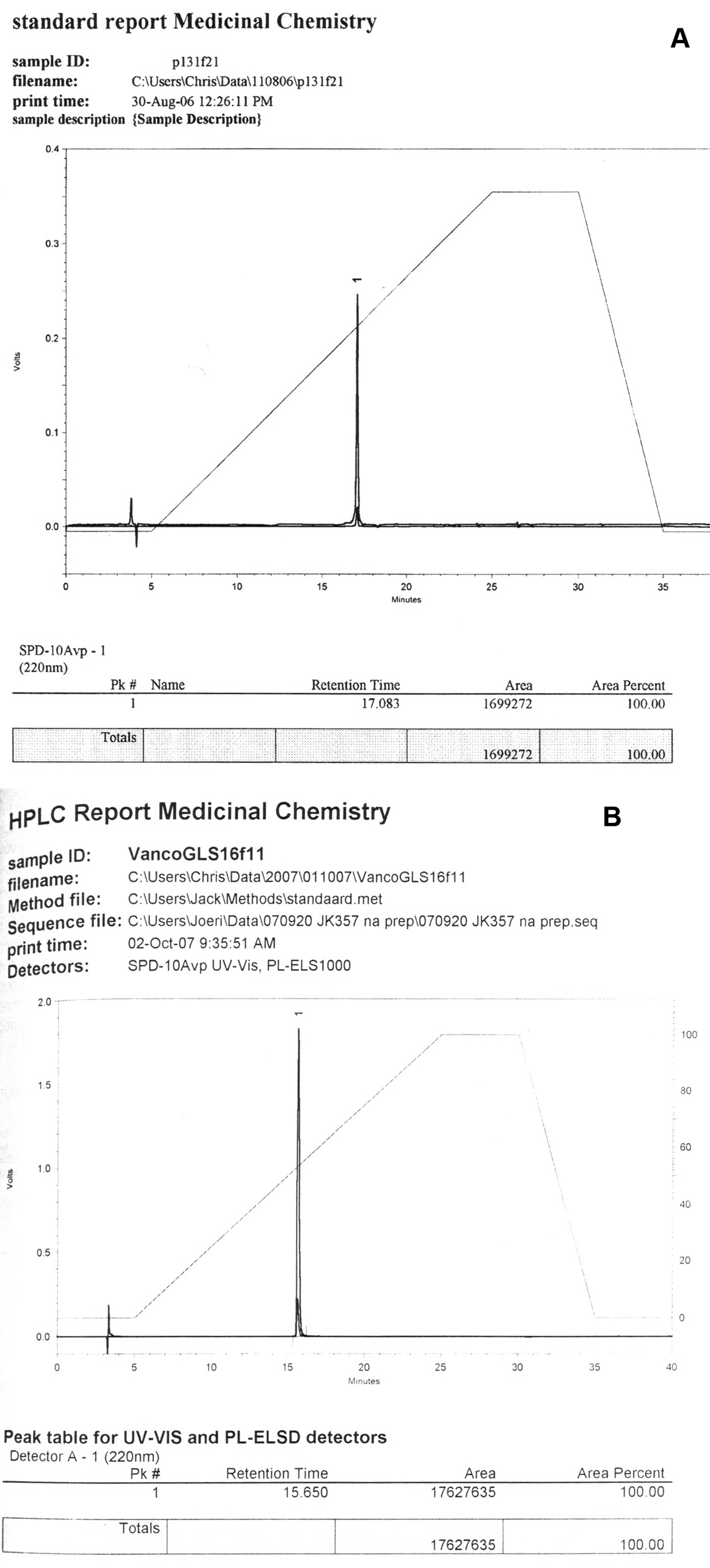

Supplement: Figure S1 — HPLC traces of vancomycin-peptide conjugates. (TIF) [file pone.0039768.s001.tif]

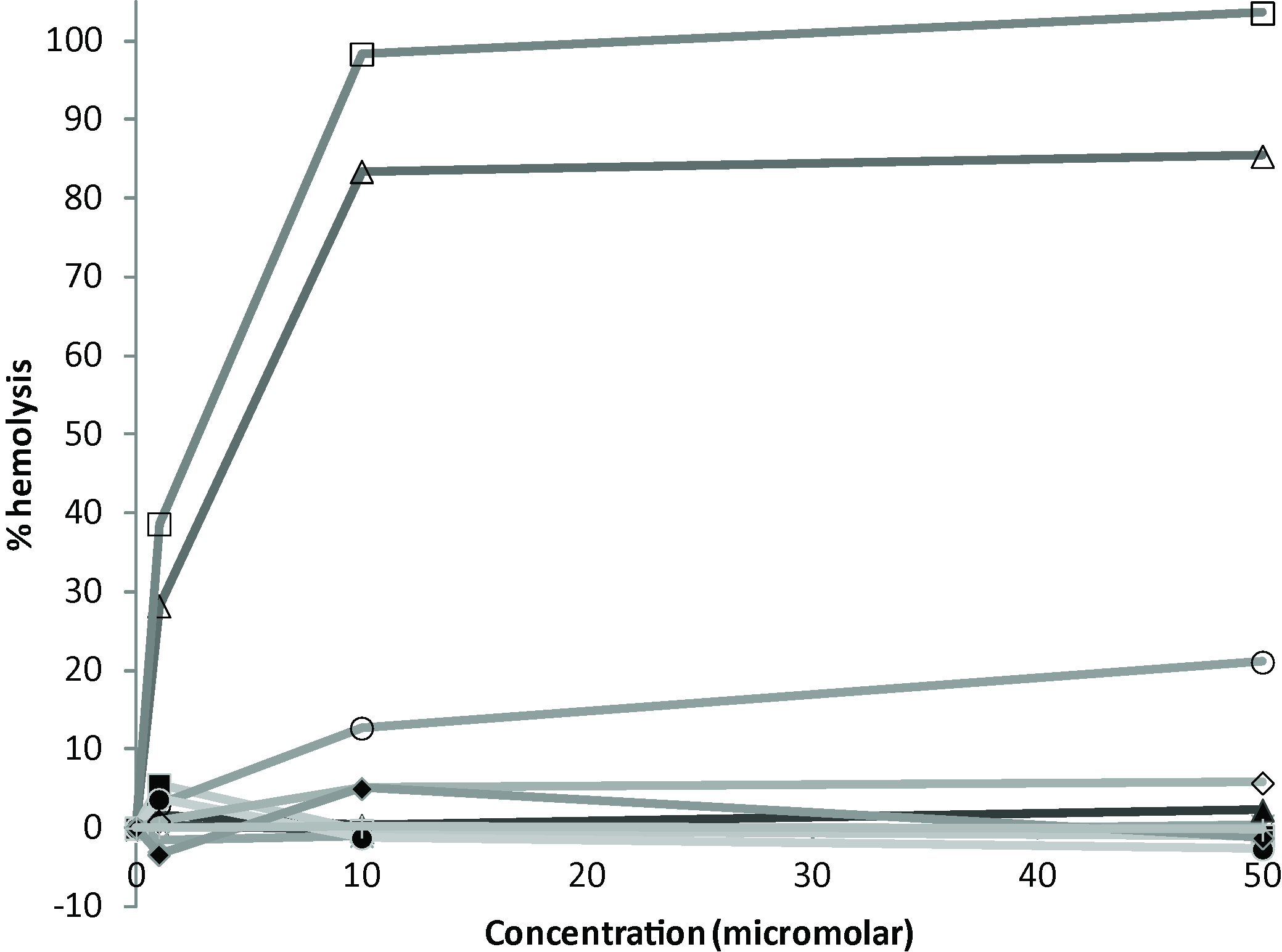

Supplement: Figure S2 — Targeted hemolysis. Erythrocytes containing lipid II (0.1%), were treated with 1 (▵), 2 (○), nisin (□), 4 (*) and 5 (⋄). Vesicles without lipid II were treated with 1 (▴), 2 (•), nisin (▪), 4 (×), 5 (⧫), and vancomycin (+). (TIF) [file pone.0039768.s002.tif]
