# Supplementary material for: Enhanced Membrane Pore Formation through High-Affinity Targeted Antimicrobial Peptides
Source: PLoS One. 2012 Jun 29;7(6):e39768. doi: 10.1371/journal.pone.0039768 (PMC3387250; doi:10.1371/journal.pone.0039768)
Supplement: Methods S1 — ΣFIC calculations for compound 2 and VRE, and synthesis of the Nisin(1-12)-peptide conjugate. (DOC) [file pone.0039768.s003.doc]

Supporting information for

**Enhanced Membrane Pore Formation through High-Affinity Targeted Antimicrobial Peptides**

Christopher J. Arnusch,^1,2#^ Roland J. Pieters,^2^* Eefjan Breukink,^1^*

^1^ Department of Membrane Biochemistry and Biophysics, Utrecht University, Utrecht, The Netherlands.

^2^ Department of Medicinal Chemistry and Chemical Biology, Utrecht University, Utrecht, The Netherlands.

^#^ Current address: Department of Desalination and Water Treatment, Ben Gurion University of the Negev, Sede Boqer Campus, Israel.

**Table S1. Minimum inhibitory concentration (MIC, µg/mL) of Nisin(1-12)-peptide derivatives.**

| Compound^a^ | V^b^ | N(1-12)**^c^** | **4** | **1** | **5** | **2** | N(1-12)-**5^d^** | |
| --- | --- | --- | --- | --- | --- | --- | --- | --- |
| VSE^e^ (15A797) | 0.5 | 128 | 256 | 16 | 128 | 4 | 64 |  |
| VRE^f^ (15A799) | 128 | 128 | 256 | 256 | 64 | 16 | 32 |  |
| *M. catarhallis* (58L028) | 32 | 256 | 64 | 64 | 8 | 32 | 16 |  |

^a^compounds tested in conjunction with compounds reported in ref. [24] ^b^V: vancomycin, values first published in ref. [24], ^c^N(1-12): nisin(1-12)*,^d^*N(1-12)-**5**: nisin(1-12)-peptide **5** conjugate. *^e^*VSE: vancomycin susceptible *Enterococci, ^f^VRE:* vancomycin resistant *Enterococci.*

**Methods S1**

Definition: Compounds are deemed to be synergistic if ΣFIC < 0.5, where

ΣFIC = FICa + FICb and FICx = MICx(in combination)/MICx(alone), where FIC is the fractional inhibitory concentration, a and b are test compounds, and x is a or b

MIC Compound a or b in combination = compound **2** . 8TFA (MW=4565) = 3.5 µM

MIC Compound a = vancomycin^.^HCl (MW=1486) = 86 µM

MIC Compound b = compound **5** . 7TFA (MW=2725) = 23.5 µM

ΣFIC = (3.5/86) + (3.5/23.5) = 0.19

*Nisin(1-12)-peptide conjugate*: The nisin(1-12) fragment bearing an alkyne group linked to the C-terminus was synthesized according to previous published procedures, using propargyl amine instead of 1-amino-3-azidopropane.[[1]](#endnote-2) Briefly, we digested nisin with trypsin and purified the nisin(1-12) fragment using preparative HPLC. This product was treated with a large excess of propargyl amine with BOP and DIPEA in a small volume of DMF for 15 min. HPLC purification afforded nisin(1-12) with the propargyl amine coupled to Nisin(1-12) fragment as a C-terminal amide. Identity was confirmed using MS. This compound, (1 mg, 0.7 µmol) and peptide-azide **5** (1.95 mg, 0.7 µmol) were dissolved in H_2_O with 0.1% DMF (400 µL). CuSO_4_^.^5H_2_O (0.8 mg, 3.6 µmol) was dissolved in H_2_O with 0.1% DMF (50 µL) and added, and subsequently NaAsc (1.4 mg, 7.1 µmol) was added in H_2_O with 0.1% DMF (40 µL). The mixture was subject to microwave heating for 10 min. at a constant temperature of 80 ^o^C. HPLC Buffer A:B (1:1) was added (1 mL), and the solution was subject to preparative HPLC. Nisin(1-12)-peptide was purified using HPLC and lyophilized for 2.2 mg of a white powder in a yield of 74 %. The identity of was confirmed with MS (Maldi *m/z,* (% abundance): 3108 (45), 3109 (90), 3110 (100), 3111 (70) 3112 (40), 3113 (30) [M + H^+^]. HPLC Retention time = 18.23 min. >98%.

1. Arnusch CJ, *et al*. (2008) The Vancomycin-Nisin(1-12) hybrid restores activity against vancomycin resistant *Enterococci.* Biochemistry 47: 12661-12663. [↑](#endnote-ref-2)
