# Supplementary material for: Enhanced Membrane Pore Formation through High-Affinity Targeted Antimicrobial Peptides
Source: PLoS One. 2012 Jun 29;7(6):e39768. doi: 10.1371/journal.pone.0039768 (PMC3387250; doi:10.1371/journal.pone.0039768)
Supplement: Table S1 — Minimum inhibitory concentration (MIC, µg/mL) of Nisin(1-12)-peptide derivatives. (DOCX) [file pone.0039768.s004.docx]

**Table S1.** Minimum inhibitory concentration (MIC, µg/mL) of Nisin(1-12)-peptide derivatives.

| Compound^a^ | V^b^ | N(1-12)**^c^** | **4** | **1** | **5** | **2** | N(1-12)-**5^d^** | |
| --- | --- | --- | --- | --- | --- | --- | --- | --- |
| VSE^e^ (15A797) | 0.5 | 128 | 256 | 16 | 128 | 4 | 64 |  |
| VRE^f^ (15A799) | 128 | 128 | 256 | 256 | 64 | 16 | 32 |  |
| *M. catarhallis* (58L028) | 32 | 256 | 64 | 64 | 8 | 32 | 16 |  |

^a^compounds tested in conjunction with compounds reported in ref. [24] ^b^V: vancomycin, values first published in ref. [24], ^c^N(1-12): nisin(1-12)*,^d^*N(1-12)-**5**: nisin(1-12)-peptide **5** conjugate. *^e^*VSE: vancomycin susceptible *Enterococci, ^f^VRE:* vancomycin resistant *Enterococci.*
